# Retrospective analysis of preoperative nutritional status and postoperative complications in gastric cancer surgery

**DOI:** 10.6026/973206300213035

**Published:** 2025-09-30

**Authors:** Navaneeth Ranjith, Sailesh Kumar, Gomes Bosco

**Affiliations:** 1 Department of Urology, Caritas Hospital, Kottayam, India; 2 Department of General Medicine, Madras Medical College, Tamil Nadu, India; 3 Department of Cardio Thoracic and Vascular Surgery (CTVS), Father Muller Medical College Hospital, Mangalore, Karnataka, India

**Keywords:** Gastric cancer, preoperative nutrition, postoperative complications, malnutrition, surgical outcomes

## Abstract

The impact of preoperative nutritional status on postoperative complications in patients undergoing gastric cancer surgery. Hence, a total of 137 patients were analyzed using objective nutritional markers including BMI, serum albumin and Nutritional Risk Screening (NRS-2002) scores. Patients with poor nutritional status experienced significantly higher rates of surgical site infection, anastomotic leak and prolonged hospital stay. Multivariate analysis confirmed malnutrition as an independent predictor of postoperative morbidity. Thus, we show the importance of early nutritional optimization in gastric cancer surgical candidates.

## Background:

According to the latest global cancer statistics, gastric cancer (GC) ranks as the fifth most common cancer worldwide and the third most prevalent in China. Surgical resection remains the primary treatment for advanced gastric cancer [1]. Preoperative nutritional status in patients with gastric cancer after surgery has attracted considerable interest. The nutritional risk index (NRI) has been widely used as a convenient and effective nutritional assessment index, but the relationship between preoperative NRI and postoperative complications in patients with gastric cancer has not been adequately studied [2]. Malnutrition is a prevalent yet often under recognized condition among patients undergoing surgery for gastric cancer, with estimates suggesting that up to 60% of such patients exhibit some degree of nutritional deficiency at diagnosis [3]. Nutritional depletion in these individuals is multifactorial, resulting from tumor-related anorexia, metabolic alterations, gastrointestinal obstruction and prior chemotherapy [4]. Compromised nutritional status adversely affects immune function, tissue repair and response to surgical stress, predisposing patients to higher rates of postoperative complications, including infections, anastomotic leaks, and delayed wound healing [5]. In the context of gastric cancer surgery, where procedures such as gastrectomy impose further metabolic and digestive burdens, preoperative nutritional optimization may be a critical determinant of postoperative outcomes [6]. Despite mounting evidence supporting nutritional screening, it is not universally integrated into preoperative protocols [7]. Therefore, it is of interest to analyze the relationship between preoperative nutritional status and the incidence of postoperative complications in gastric cancer patients, thereby emphasizing the role of early nutrition assessment in surgical planning and risk stratification.

## Methods:

This retrospective analysis was conducted at a high-volume tertiary cancer center and included 137 patients who underwent curative gastric cancer surgery between January 2022 and December 2023. Inclusion criteria were adult patients aged 18-80 years with histologically confirmed gastric adenocarcinoma undergoing subtotal or total gastrectomy. Patients with incomplete records, emergency surgeries, or pre-existing infections were excluded. Preoperative nutritional status was assessed using body mass index (BMI), serum albumin levels, and Nutritional Risk Screening (NRS-2002) scores extracted from patient records within 7 days prior to surgery. Postoperative complications occurring within 30 days of surgery were recorded, including surgical site infection (SSI), anastomotic leak, pneumonia, delayed gastric emptying, and reoperation. Patients were stratified into nutritionally at-risk (NRS ≥3 and/or albumin <3.5 g/dL) and nutritionally adequate groups. Statistical analysis was performed using chi-square and t-tests to compare complication rates between groups. Multivariate logistic regression was used to identify independent predictors of complications. A p-value <0.05 was considered statistically significant.

## Results:

Out of 137 patients analyzed, 58 (42.3%) were identified as nutritionally at-risk based on NRS-2002 scores ≥3 and/or serum albumin <3.5 g/dL. These patients experienced significantly higher rates of postoperative complications including infections, anastomotic leaks, and prolonged hospital stays. Nutritional risk remained a significant independent predictor of complications after adjustment for age, BMI, and comorbidities. Table 1 (see PDF) shows Patients in the nutritionally at-risk group had significantly lower BMI and serum albumin compared to the nutritionally adequate group. Table 2 (see PDF) shows there was a higher incidence of overall postoperative complications in the nutritionally at-risk group. Table 3 (see PDF) shows surgical site infections were significantly more common in malnourished patients. Table 4 (see PDF) shows Anastomotic leaks were also significantly more frequent in the at-risk group. Table 5 (see PDF) shows Pneumonia incidence was elevated in patients with poor nutritional status. Table 6 (see PDF) shows there was a statistically significant increase in delayed gastric emptying among the at-risk group. Table 7 (see PDF) shows Hospital stay was longer in nutritionally at-risk patients. Table 8 (see PDF) shows ICU admissions were more frequent in the at-risk group, indicating more severe complications. Table 9 (see PDF) shows Reoperation rates were higher in the nutritionally compromised group. Table 10 (see PDF) shows Mortality, although low, occurred only in the at-risk group.

## Discussion:

This retrospective study confirms a strong association between poor preoperative nutritional status and increased postoperative complications in patients undergoing gastric cancer surgery. Patients identified as nutritionally at-risk defined by serum albumin <3.5 g/dL and/or NRS-2002 score ≥3 had significantly higher rates of surgical site infections, anastomotic leaks, pneumonia, delayed gastric emptying, ICU admissions and reoperations [8]. Furthermore, they had longer hospital stays and lower patient satisfaction, indicating a broad impact of nutritional deficiency on surgical recovery and quality of life [9]. These findings are consistent with existing literature highlighting the detrimental effects of malnutrition on immune competence, tissue repair and metabolic resilience [10]. Hypoalbuminemia, a surrogate marker of both protein deficiency and systemic inflammation, emerged as an independent predictor of complications [11]. The elevated mortality seen exclusively in the at-risk group, though low in absolute numbers, underscores the potential severity of compromised nutritional status [12]. Importantly, multivariate analysis revealed that nutritional markers were stronger predictors of complications than age or BMI, emphasizing the need to incorporate objective nutritional screening into routine preoperative assessment [13]. While BMI is often used as a superficial nutritional index, our data show it lacked significant predictive power compared to albumin or NRS-2002 scores [14].

This study adds to growing evidence advocating for early nutritional intervention. Prehabilitation strategies-including enteral supplementation, dietary counseling, and immunonutrition may improve surgical outcomes, particularly in resource-limited settings where delays in cancer surgery are common. Limitations include its retrospective design, potential variability in surgical technique, and reliance on documentation for nutritional scoring. Nonetheless, the consistent and statistically significant trends across multiple outcomes support the integration of nutritional optimization into gastric cancer surgical pathways.

## Conclusion:

Poor preoperative nutritional status significantly increases the risk of postoperative complications in gastric cancer surgery. Serum albumin and NRS-2002 scores are reliable predictors of surgical outcomes. Routine nutritional screening and early intervention should be integrated into preoperative protocols to enhance recovery and reduce morbidity.

## References

[1] Liao Y (2025). Nutrition..

[2] Zou Y (2025). Front Oncol..

[3] Hsu PI (2021). Journal of Formos Med Assoc..

[4] Kanda M (2020). Surg Today..

[5] Kubota T (2020). Ann Gastroenterol Surg..

[6] Fang Z (2021). Expert Rev Gastroenterol Hepatol..

[7] Hirahara N (2021). Surg Endosc..

[8] Tegels JJ (2014). Journal of Gastrointest Surg..

[9] Abe A (2020). Oral Dis..

[10] Loan BTH (2018). Nutrition..

[11] Sugawara K (2020). Eur J Surg Oncol..

[12] Deftereos I (2020). Eur J Surg Oncol..

[13] Zhao B (2018). Nutr Cancer..

[14] Takagi K (2019). BMC Surg..

